# Whole-exome sequencing identified a novel mutation of AURKC in a Chinese family with macrozoospermia

**DOI:** 10.1007/s10815-018-1374-3

**Published:** 2018-12-29

**Authors:** Juan Hua, Yang-yang Wan

**Affiliations:** 1 Department of Biochemistry and Molecular Biology, School of Basic Medical Sciences, Anhui Medical University, Hefei, 230032 China; 2 Center for Reproductive Medicine, Anhui Provincial Hospital Affiliated to University of Science and Technology of China, Hefei City, Anhui Province China

**Keywords:** Macrozoospermia, AURKC, Assisted reproduction technologies, Whole-exome sequencing

## Abstract

**Purpose:**

Macrozoospermia is a rare sperm morphologic abnormality associated with male infertility and is characterized by a high percentage of spermatozoa with large irregular heads. The aim of this study was to identify the genetic cause of an infertile male with macrozoospermia from a consanguineous family.

**Methods:**

Whole-exome sequencing (WES) was performed using peripheral blood genomic DNA from the patient and his parents.

**Results:**

WES analysis of the patient with macrozoospermia from a consanguineous family allowed the identification of a novel homozygous missense variant in the AURKC gene (c.269G>A). Bioinformatics analysis also suggested this variant a pathogenic mutation. Quantitative real-time PCR analysis showed that the mRNA level of AURKC is significantly decreased in the patient compared with his father. Moreover, no embryos were available for transfer after ICSI.

**Conclusions:**

These results further support the important role of AURKC in male infertility and guide the practitioner in optimal decision making for patients with macrozoospermia.

**Electronic supplementary material:**

The online version of this article (10.1007/s10815-018-1374-3) contains supplementary material, which is available to authorized users.

## Introduction

Approximately 70 million couples worldwide suffer from infertility [1], and approximately half of these cases are due to male factors [2]. Macrozoospermia is a rare sperm morphologic abnormality associated with male infertility and is characterized by a high percentage of spermatozoa with large irregular heads [3, 4]. This syndrome, first reported in 1977, affects < 1% of infertile men [3]. It is considered to be an autosomal recessive type of teratozoospermia that results in male infertility [5]. Currently, the most relevant single-gene defect that has been identified in a patient with macrozoospermia is a mutation in Aurora Kinase C (*AURKC*) [5–7].

The *AURKC* gene encodes a member of a highly conserved serine/threonine kinase family, which plays crucial roles in centrosome function, homologous chromosome segregation, and cytokinesis during meiosis [7, 8]. Until now, only five mutations of *AURKC* have been described to be associated with macrozoospermia: c.144delC (p.L499Wfs22), c.744C>G (p.Y248*), c686G>A (p.C229Y), c.930+38G>A (occurs in the 3′-UTR), and c.436-2A>G (splicing site mutation that leads to the skipping of exons 5) [6–13]. These mutations in humans lead to reduced protein function, which results in meiotic failure, but spermatogenesis is not affected, leading to the production of large-headed spermatozoa [9, 10, 14].

Here, we report a novel homozygous missense variant in the *AURKC* gene in an infertile male from a consanguineous family identified via whole-exome sequencing. In addition, this variant has a high probability of pathogenicity according to in silico analysis. ICSI was also performed and failed to generate any embryos suitable for transfer. These results further support the important role of *AURKC* in male infertility and guide the practitioner in optimal decision-making for patients with macrozoospermia.

## Materials and methods

### Patient

**Fig. 1:**
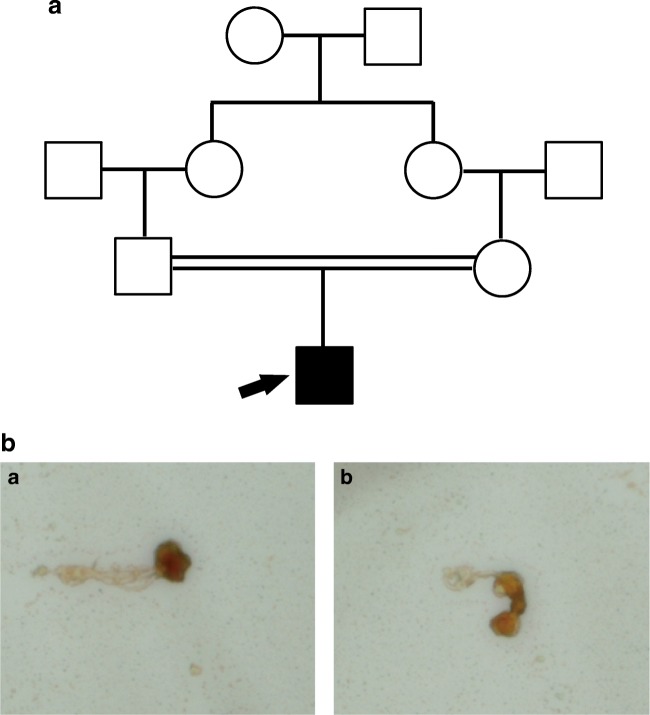
A patient with macrozoospermia in a consanguineous family. **A** Family tree of the patient. **B** Large-headed spermatozoa were observed by light microscopy (a and b)

The proband was a 27-year-old treated for infertility at Anhui Provincial Hospital. The results of the patient’s semen tests are described in Table 1. The results revealed that the patient suffered from macrozoospermia (close to 100% large-headed spermatozoa with a sperm count of 1 M/ml) (Table 1). The parents of the patient were first-degree cousins (Fig. 1). The patient exhibited normal erection and ejaculation, and reported having regular sexual intercourse 2–3 times/week; however, his wife had been unable to get pregnant since they were married in 2015. The patient had no history of unhealthy activity or contact with adverse chemicals. Physical examination revealed normally developed male external genitalia, normal bilateral testicular size, and no abnormality in the bilateral spermatic veins upon palpation. The proband did not have primary microcephaly or respiratory disease. The chromosomal karyotype of the patient was normal (46; XY), and no deletion was found in the Y chromosome. The patient hormone levels were normal.

**Table 1:** Sperm parameters and morphology on ejaculate from patient

Sperm characteristics	Patient	Reference values
Sperm volume (ml)	1.8	≥ 1.5
Number spz × 10^6^ per ml	1.71	≥ 15
Large-headed (%)	96	–
Sperm tail anomaly (%)	83	–
Motility (%) (a b, 1 h)	7.69	≥ 30
Viability (%)	15.4	≥ 55
Normal spz (%)	4%	≥ 15

*Spz*, spermatozoa

This project was approved by the ethics committee of Anhui Medical University. All study members gave their written informed consent before sample collection.

### Genomic DNA extraction and whole-exome sequencing (WES)

The genomic DNA from the patient and his parents was extracted from peripheral blood samples using a QIAamp DNA blood midi kit (Qiagen, Hilden, Germany) according to the manufacturer’s protocol.

Genomic DNA from the proband and his parents was subjected to WES. WES was performed by the WuXi NextCODE in Shanghai on a HiSeq2000 sequencing platform (Illumina, San Diego, CA, USA). After the adaptors were removed, the WES raw reads were aligned to the reference genome Hg19 using the Burrows-Wheeler aligner, followed by removal of PCR duplicates. Variants including single-nucleotide polymorphisms and indels were identified using SAMtools and annotated by ANNOVAR software. A candidate gene was considered a variant that fulfilled the following criteria: (i) missense, nonsense, frame shift, and splice site variants, (ii) absent or rare (frequency below 1%) in the two databases (dbSNP, 1000G), and (iii) homozygous variants in the patient and heterozygous variants in his parents.

### Sanger sequencing validation

The mutation in AURKC in the proband and his parents was validated using Sanger sequencing. We amplified the PCR products of exon 3 of the AURKC gene using specific primes (the forward primer is 5′-AACCAGGATTCGAGTGTCTG-3′, and the reverse primer is 5′-CAATCTCCAGGTAGACGATGGAG-3′). Then, the PCR products were sequenced on an ABI 3730XL automated sequencer (Applied Biosystems, Forster City, CA, USA).

### RNA extraction and Q-PCR

RNA extraction was carried out on the whole blood using TRI REAGENT®BD (Molecular Research Center) using the manufacturer’s protocol. Reverse transcription was performed in patient and his parents with 5 μl of extracted RNA. Hybridization of the oligo-dT was performed by incubating for 30 min at 42 °C and quenching on ice with the following mix: 5 μl of RNA, 10 μl of 2Xsupermix (10 mM, Pharmacia), 1 μl of gDNA (0.5 mM, Roche diagnostics), and 4 μl of H_2_O. Then 2 μl of the obtained cDNA mix was used for the quantitative PCR (Q-PCR) using a StepOne-PlusTM Real-Time PCR System (Life Technologies) with Power SYBR Green PCR Master Mix (Life Technologies) according to the manufacturer’s protocol. Quantification of the fold change in gene expression was determined by the relative quantification method (2^−ΔΔCT^) using the *gapdh* gene as a reference. Data are shown as the average fold increase standard error of the mean. Primers are described in supplementary file 1.

### ICSI, embryo, and evaluation of embryo quality

The patient and his wife underwent intracytoplasmic sperm injection (ICSI) at Anhui Provincial Hospital. Briefly, embryo culture was performed using Vitrolife G-SERIES™ culture media (Vitrolife, Goteborg, Sweden) according to the manufacturer’s instructions. The patient’s wife had undergone one ICSI cycle. Detailed results are described in the “Results” section.

### Sequence alignment of AURKC protein

Sequence alignment of the AURKC protein in different species was conducted using ClustalX2.1. The number of each species was as follows: *Homo sapiens* (NP_001015878.1), *Mus musculus* (AAI00338.1), *Bos Taurus* (NP_001180124.1), *Desmodus rotundus* (XP_024425801.1), *Pan paniscus* (XP_003816716.1), *Pongo abelii* (XP_002829903.1), and *Rattus norvegicus* (NP_001295465.1).

## Results

### WES analysis of a patient with macrozoospermia

**Fig. 2:**
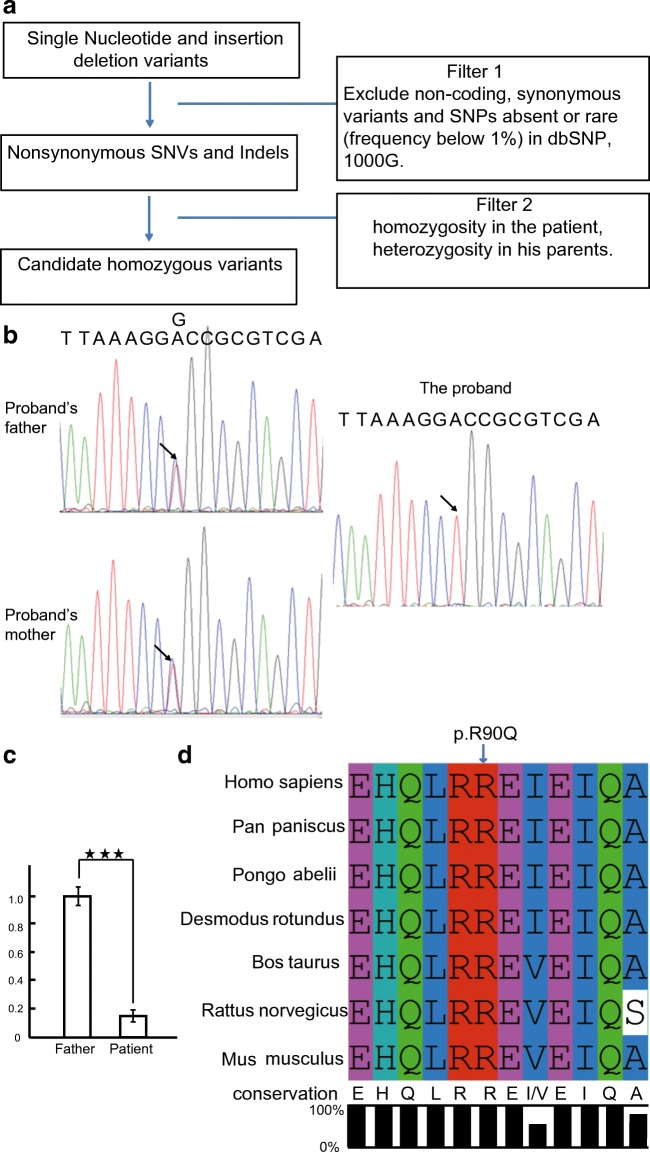
Whole-exome sequencing analysis of the consanguineous family. **A** Filter strategies used in this study. **B** Sanger sequencing validation of the affected patient and his parents. The arrow indicates the mutation site. **C**Q-PCR analysis of mRNA expressions of *AURKC* in the patient and his father. Messenger RNA expression determined by Q-PCR is calculated as a ratio relative to *gapdh* and expressed relative to his father. **D** Sequence alignment of AURKC protein in different species. The arrow refers to the mutation site

To identify the genetic cause of the macrozoospermia, we performed WES using peripheral blood genomic DNA from the patient and his parents to identify the putative pathogenic mutation. Considering the family history of consanguinity, we focused on homozygous mutations. After of the exclusions of frequent variants and application of technical and biological filters (see “Materials and methods” section and Fig. 2A), a limited list of homozygous mutations was established (Supplementary Table 1). To determine whether any of these 36 genes may be related to macrozoospermia, we first examined the tissue expression patterns for each gene. Among these homozygous variant genes, only two genes exhibited testis-enriched expression (Fig. S1). One of the identified genes was AURKC, and a new mutation (c.269G>A, GenBank accession number, NM_001015878) in exon 3 was identified in the patient by WES. Accordingly, the change in amino acids was determined to be Arg90Gln. Another identified gene was gametogenetin (*GGN*), and a mutation (c.148T>C, GenBank accession number, NM_152657.3) in exon 3 was identified in the patient by WES. Accordingly, the change in amino acids was determined to be Trp50Arg.

### Validation by Sanger sequencing

We next performed Sanger sequencing to verify the homozygous variant in the *AURKC* and *GGN* genes in both the patient and his parents. Exon 3 of the *AURKC* gene was amplified from genomic DNA of the patient and his parents using PCR. The homozygous missense variant in *AURKC* was validated in the patient, and his parents had heterozygous alleles (Fig. 2B).

### Detrimental effects of the identified variant in *AURKC*

Unfortunately, we could not get access to testicular biopsy samples from the affected patient and his parents; therefore, we set up Q-PCR to study the effect of the variation. Q-PCR analysis demonstrated that mRNA levels of AURKC were down-regulated significantly in the patient (Fig. 2C).

### In silico analysis of the mutation

Bioinformatics analysis of the c.269G>A mutation in the AURKC gene by three online pathogenicity prediction tools (Polyphen-2, SIFT, Mutation taster) suggested that this mutation is most likely a disease-causing mutation (Supplementary Table 2). This variant is a novel mutation that was absent in the ExAC, 1000G, and gnomAD databases. The mutation site at R90 was highly conserved from human to zebrafish, indicating an important role of this site for the function of AURKC protein (Fig. 2D).

### ICSI using the patient’s sperm

After centrifugation on density gradients and careful examination, a few “normal-looking” spermatozoa that fit into an ICSI micropipette were selected. One ICSI cycle was attempted for the patient at Anhui Provincial Hospital. For the ICSI cycles, we collected 10 eggs and 7 MII oocytes; none of the embryos developed to the blastocyst stage.

## Discussion

In the last decade, five mutations (c.144delC (p.L49Wfs22), c.744C>G (p.Y248*), c686G>A (p.C229Y), c.930+38G>A (occurs in the 3′-UTR), and c.436-2A>G (splicing site mutation that leads to the skipping of exons 5)) [8–13] in the *AURKC* gene have been reported to be associated with macrozoospermia. In this study, we identified a novel homozygous mutation (c.269 G>A; p.R90Q) in the *AURKC* gene in a macrozoocephalic patient from a consanguineous family. One ICSI cycle was carried out in the patient, but none of the embryos developed to the blastocyst stage.

To date, numerous reports have provided evidence suggesting that failures in chromosome segregation and/or cytokinesis during the first, second, or both meiotic divisions are the major cause of macrozoospermia [15–19]. Aurora kinases are highly evolutionarily conserved kinases that are required for accurate chromosome segregation and cytokinesis during meiosis [20]. *AURKC* has 7 exons and encodes a 309 amino acid protein in humans. *AURKC* is a component of the Aurora kinase family and is predominantly expressed in the testis [21, 22]. In this study, we found a new missense mutation in exon 3 of the *AURKC* gene that led to an amino acid change (p.R90Q). The sequence alignment of the AURKC protein showed that this mutation site is conserved among different species (Fig. 2C). We utilized three online pathogenicity predict tools (Polyphen-2, SIFT, Mutation taster) to predict the harmfulness of this variant (Table 2). The results suggested that this variant has a high probability of pathogenicity.

Previous studies have compared the values of a routine spermogram and spermocytogram between patients with and without an *AURKC* mutation [8]. The results showed that the proportions of large-headed spermatozoa generally reached far higher values, and the presence of 1% normal spermatozoa is the most discriminant parameter in patients with *AURKC* mutations [8]. Consistent with previous studies, the patient with missense variant in the *AURKC* gene (c.269G>A) in this study showed 96% large-headed spermatozoa. However, the proportions of normal spermatozoa reached 4%. One explanation could be that in addition to macrozoospermia, this patient studied here also has a low sperm number (1.71 × 10^6^). Variable scoring of the normal spermatozoa features between operators and laboratories could also influence the results as some spermatozoa with a slight morphology defect in head or flagella could be considered normal [8].

Numerous publications have described a failure of pregnancy in patients with macrozoospermia [5, 7, 12, 16–18, 23–25]. In addition, further studies have recommended that systematic genetic screening of the AURKC gene be performed when a patient has more than 30% enlarged head spermatozoa. If a mutation in the *AURKC* gene is found, ICSI should not be attempted [7, 26]. In this study, the patient’s wife failed to become pregnant after one ICSI cycle, again demonstrating the correlation between *AURKC* gene mutation and ICSI outcome. Consequently, ICSI in such a patient is not recommended.

In conclusion, we identified a novel missense mutation (c.269 G>A; p.R90Q) in the AURKC gene. To date, this is the sixth reported variant in the AURKC gene associated with macrozoospermia. This study expands the spectrum of AURKC mutations and helps guide the practitioners to make optimal decisions for patients with macrozoospermia.

## Electronic supplementary material

Fig S1 (PDF 405 kb)

Table S1 (PDF 382 kb)

Table S2 (PDF 283 kb)

Supplementary File 1 (XLS 26 kb)

## References

[1] Boivin J, Bunting L, Collins JA, Nygren KG (2007). International estimates of infertility prevalence and treatment-seeking: potential need and demand for infertility medical care. Hum Reprod.

[2] Silber SJ (2000). Evaluation and treatment of male infertility. Clin Obstet Gynecol.

[3] Nistal M, Paniagua R, Herruzo A (1977). Multi-tailed spermatozoa in a case with asthenospermia and teratospermia. Virchows Arch B Cell Pathol.

[4] Brahem S, Mehdi M, Elghezal H, Saad A (2012). Study of aneuploidy rate and sperm DNA fragmentation in large-headed, multiple-tailed spermatozoa. Andrologia.

[5] Ghedir H, Gribaa M, Mamai O, Ben Charfeddine I, Braham A, Amara A, Mehdi M, Saad A, Ibala-Romdhane S (2015). Macrozoospermia: screening for the homozygous c.144delC mutation in AURKC gene in infertile men and estimation of its heterozygosity frequency in the Tunisian population. J Assist Reprod Genet.

[6] Dieterich K, Soto Rifo R, Faure AK, Hennebicq S, Ben Amar B, Zahi M, Perrin J, Martinez D, Sele B, Jouk PS (2007). Homozygous mutation of AURKC yields large-headed polyploid spermatozoa and causes male infertility. Nat Genet.

[7] Ben Khelifa M, Zouari R, Harbuz R, Halouani L, Arnoult C, Lunardi J, Ray PF (2011). A new AURKC mutation causing macrozoospermia: implications for human spermatogenesis and clinical diagnosis. Mol Hum Reprod.

[8] Ben Khelifa M, Coutton C, Blum MG, Abada F, Harbuz R, Zouari R, Guichet A, May-Panloup P, Mitchell V, Rollet J (2012). Identification of a new recurrent aurora kinase C mutation in both European and African men with macrozoospermia. Hum Reprod.

[9] Dieterich K, Zouari R, Harbuz R, Vialard F, Martinez D, Bellayou H, Prisant N, Zoghmar A, Guichaoua MR, Koscinski I, Kharouf M, Noruzinia M, Nadifi S, Sefiani A, Lornage J, Zahi M, Viville S, Sele B, Jouk PS, Jacob MC, Escalier D, Nikas Y, Hennebicq S, Lunardi J, Ray PF (2009). The Aurora Kinase C c.144delC mutation causes meiosis I arrest in men and is frequent in the North African population. Hum Mol Genet.

[10] Harbuz R, Zouari R, Dieterich K, Nikas Y, Lunardi J, Hennebicq S, Ray PF (2009). Function of aurora kinase C (AURKC) in human reproduction. Gynecol Obstet Fertil.

[11] El Kerch F, Lamzouri A, Laarabi FZ, Zahi M, Ben Amar B, Sefiani A (2011). Confirmation of the high prevalence in Morocco of the homozygous mutation c.144delC in the aurora kinase C gene (AURKC) in the teratozoospermia with large-headed spermatozoa. J Gynecol Obstet Biol Reprod.

[12] Ounis L, Zoghmar A, Coutton C, Rouabah L, Hachemi M, Martinez D, Martinez G, Bellil I, Khelifi D, Arnoult C, Fauré J, Benbouhedja S, Rouabah A, Ray PF (2015). Mutations of the aurora kinase C gene causing macrozoospermia are the most frequent genetic cause of male infertility in Algerian men. Asian J Androl.

[13] Eloualid A, Rouba H, Rhaissi H, Barakat A, Louanjli N, Bashamboo A, McElreavey K (2014). Prevalence of the Aurora kinase C c.144delC mutation in infertile Moroccan men. Fertil Steril.

[14] Fellmeth JE, Ghanaim EM, Schindler K (2016). Characterization of macrozoospermia-associated AURKC mutations in a mammalian meiotic system. Hum Mol Genet.

[15] Escalier D, Bermudez D, Gallo JM, Viellefond A, Schrevel J (1992). Cytoplasmic events in human meiotic arrest as revealed by immunolabelling of spermatocyte proacrosin. Differentiation.

[16] In't Veld PA, Broekmans FJ, de France HF, Pearson PL, Pieters MH, van Kooij RJ (1997). Intracytoplasmic sperm injection (ICSI) and chromosomally abnormal spermatozoa. Hum Reprod.

[17] Weissenberg R, Aviram A, Golan R, Lewin LM, Levron J, Madgar I, Dor J, Barkai G, Goldman B (1998). Concurrent use of flow cytometry and fluorescence in-situ hybridization techniques for detecting faulty meiosis in a human sperm sample. Mol Hum Reprod.

[18] Benzacken B, Gavelle FM, Martin-Pont B, Dupuy O, Lievre N, Hugues JN, Wolf JP (2001). Familial sperm polyploidy induced by genetic spermatogenesis failure: case report. Hum Reprod.

[19] Devillard F, Metzler-Guillemain C, Pelletier R, DeRobertis C, Bergues U, Hennebicq S, Guichaoua M, Sele B, Rousseaux S (2002). Polyploidy in large-headed sperm: FISH study of three cases. Hum Reprod.

[20] Yang KT, Tang CJ, Tang TK (2015). Possible role of Aurora-C in meiosis. Front Oncol.

[21] Bernard M, Sanseau P, Henry C, Couturier A, Prigent C (1998). Cloning of STK13, a third human protein kinase related to Drosophila aurora and budding yeast Ipl1 that maps on chromosome 19q13.3-ter. Genomics.

[22] Tang CJ, Lin CY, Tang TK (2006). Dynamic localization and functional implications of Aurora-C kinase during male mouse meiosis. Dev Biol.

[23] Yurov YB, Saias MJ, Vorsanova SG, Erny R, Soloviev IV, Sharonin VO, Guichaoua MR, Luciani JM (1996). Rapid chromosomal analysis of germ-line cells by FISH: an investigation of an infertile male with large-headed spermatozoa. Mol Hum Reprod.

[24] Lewis-Jones I, Aziz N, Seshadri S, Douglas A, Howard P (2003). Sperm chromosomal abnormalities are linked to sperm morphologic deformities. Fertil Steril.

[25] Perrin A, Morel F, Moy L, Colleu D, Amice V, De Braekeleer M (2008). Study of aneuploidy in large-headed, multiple-tailed spermatozoa: case report and review of the literature. Fertil Steril.

[26] Carmignac V, Dupont JM, Fierro RC, Barberet J, Bruno C, Lieury N, Dulioust E, Auger J, Fauque P (2017). Diagnostic genetic screening for assisted reproductive technologies patients with macrozoospermia. Andrology.

