# Supplementary figures and images for: Whole-exome sequencing identified a novel mutation of AURKC in a Chinese family with macrozoospermia
Source: J Assist Reprod Genet. 2018 Dec 29;36(3):529–34. doi: 10.1007/s10815-018-1374-3 (PMC6439091; doi:10.1007/s10815-018-1374-3)

Supplementary Figure 1

A

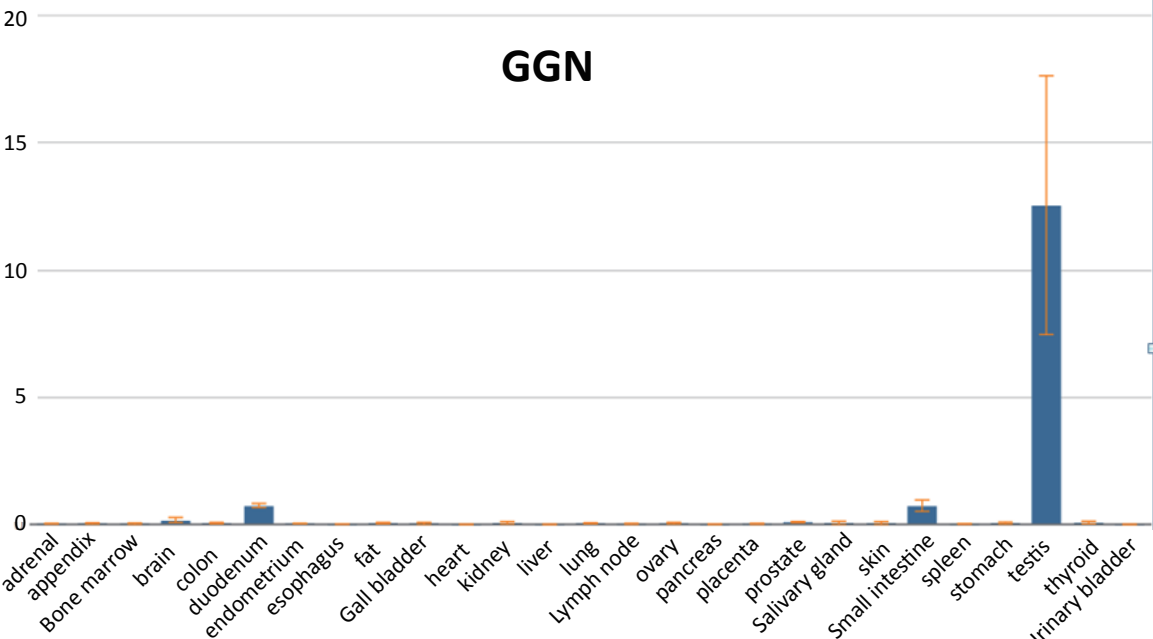

B

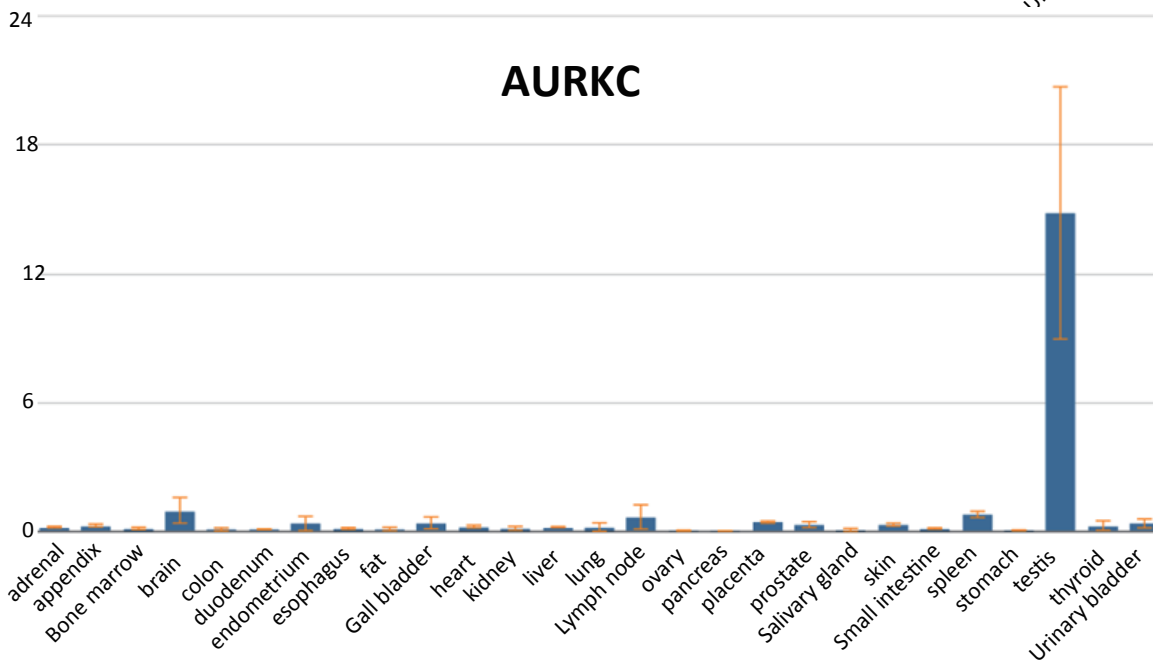

Supplement: Supplementary file 1 — (PDF 405 kb) [file 10815_2018_1374_MOESM1_ESM.pdf]
