# Supplementary material for: Whole-exome sequencing identified a novel mutation of AURKC in a Chinese family with macrozoospermia
Source: J Assist Reprod Genet. 2018 Dec 29;36(3):529–34. doi: 10.1007/s10815-018-1374-3 (PMC6439091; doi:10.1007/s10815-018-1374-3)
Supplement: Supplementary file 2 — (PDF 382 kb) [file 10815_2018_1374_MOESM2_ESM.pdf]

Supplementary Table 1. Homozygous sequence variants were found by whole-exome sequencing.

Gene	Change	Annotation
AMPD2	missense variant	NM_004037.7:c.1300A>G (p.Ile434Val)
CD58	missense variant	NM_001779.2:c.278A>G (p.Tyr93Cys)
SRGAP2C	missense variant	NM_001271872.1:c.281A>G (p.His94Arg)
VPS45	missense variant	NM_007259.4:c.506T>G (p.Leu169Arg)
FLG	missense variant	NM_002016.1:c.3397C>T (p.Arg1133Trp)
KCNN3	inframe insertion	NM_001204087.1:c.230_241dupAGCAGCAGCAGC (p.Gln77_Gln80dup)
USH2A	splice region variant	NM_206933.2:c.848+5G>C
ZNF678	missense variant	NM_178549.3:c.853A>T (p.Ile285Phe)
SNAP47	splice region variant	NM_053052.3:c.1249-6C>T
C1orf35	missense variant	NM_024319.2:c.265A>C (p.Lys89Gln)
IBA57	missense variant	NM_001010867.2:c.947A>C (p.Asn316Thr)
HIST3H3	missense variant	NM_003493.2:c.190C>T (p.Arg64Cys)
CCSAP	missense variant	NM_145257.3:c.311A>G (p.Gln104Arg)
TARBP1	missense variant	NM_005646.3:c.2968A>C (p.Asn990His)
AURKC	missense variant	NM_001015878.1:c.269G>A (p.Arg90Gln)
ARHGAP21	missense variant	NM_020824.3:c.5654C>T (p.Thr1885Met)
PDSS1	missense variant	NM_014317.3:c.407T>G (p.Phe136Cys)
PCDH15	missense variant	NM_001142763.1:c.3466G>A (p.Gly1156Arg)
ASCL1	inframe insertion	NM_004316.3:c.178_186dupCAGCAGCAG (p.Gln60_Gln62dup)
FBN3	missense variant	NM_032447.3:c.5857C>T (p.Arg1953Cys)
MYO1F	missense variant	NM_012335.3:c.2123T>A (p.Val708Glu)
MUC16	missense variant	NM_024690.2:c.29510C>T (p.Thr9837Ile)
MRI1	missense variant	NM_001031727.2:c.830A>G (p.Tyr277Cys)
GGN	missense variant	NM_152657.3:c.148T>C (p.Trp50Arg)
DYRK1B	missense variant	NM_004714.1:c.1031G>A (p.Arg344His)
C19orf54	missense variant	XM_005258778.1:c.16C>G (p.Leu6Val)
CD79A	missense variant	NM_001783.3:c.301G>A (p.Gly101Arg)
CEACAM16	splice region variant	NM_001039213.2:c.37+7G>A
ZNF577	stop gained	NM_032679.2:c.193C>T (p.Arg65Ter)
CACNG6	missense variant	NM_145814.1:c.365C>T (p.Thr122Met)
ZSCAN18	missense variant	NM_001145542.1:c.1637C>T (p.Ala546Val)
PJA2	missense variant	NM_014819.4:c.631G>A (p.Ala211Thr)
SLC22A23	splice region variant	NM_015482.1:c.1578A>G (NM_015482.1:c.1578A>G(p.%3D))
IP6K3	missense variant	NM_054111.4:c.514A>G (p.Asn172Asp)
ZNF318	missense variant	NM_014345.2:c.1970G>C (p.Cys657Ser)
GCM1	missense variant	NM_003643.3:c.34G>A (p.Glu12Lys)
