# Supplementary material for: Whole-exome sequencing identified a novel mutation of AURKC in a Chinese family with macrozoospermia
Source: J Assist Reprod Genet. 2018 Dec 29;36(3):529–34. doi: 10.1007/s10815-018-1374-3 (PMC6439091; doi:10.1007/s10815-018-1374-3)
Supplement: Supplementary file 3 — (PDF 283 kb) [file 10815_2018_1374_MOESM3_ESM.pdf]

Table 2 In silico analysis of the 269 G>A mutation in AKRUC

Mutation	Amino acid change	Polyphen-2[a]	SIFT[b]	Mutation taster[c]	ExAC	1000G	gnomAD
c.G269A	p.R90Q	possible damaging (0.651)	affect protein function (0.00)	diease causing (0.9999)	0	0	0

a Polyphen-2 (http://genetics.bwh.harvard.edu/pph2/).
b SIFT (http://gsift.jcvi.org).
c Mutation taster (http://www.mutationtaster.org/)
